# A Complex Relationship between Quality of Life, Anxiety, and Depression among General Population during Second Year of COVID-19 Pandemic: A Population-Based Study

**DOI:** 10.3390/jcm13133874

**Published:** 2024-06-30

**Authors:** Danijela Radulovic, Srdjan Masic, Dejana Stanisavljevic, Dejan Bokonjic, Svetlana Radevic, Nina Rajovic, Nikola V. Milić, Ivana Simic Vukomanovic, Biljana Mijovic, Maja Vukovic, Milena Dubravac Tanaskovic, Mirka Jojic, Jelena Vladicic Masic, Dragan Spaic, Natasa M. Milic

**Affiliations:** 1 Department of Primary Health Care and Public Health, Faculty of Medicine Foca, University of East Sarajevo, 73300 Foca, Bosnia and Herzegovina; 2 Institute for Medical Statistics and Informatics, Faculty of Medicine, University of Belgrade, 11000 Belgrade, Serbia; 3 Department of Pediatrics, Faculty of Medicine Foca, University of East Sarajevo, 73300 Foca, Bosnia and Herzegovina; 4 Department of Social Medicine, Faculty of Medical Sciences, University of Kragujevac, 34000 Kragujevac, Serbia; 5 Department of Internal Medicine, Faculty of Medicine Foca, University of East Sarajevo, 73300 Foca, Bosnia and Herzegovina; 6 Department of Internal Medicine, Division of Nephrology and Hypertension, Mayo Clinic, Rochester, MN 55902, USA

**Keywords:** mental health, depression, anxiety, quality of life, COVID-19 pandemic, general population, Republic of Srpska

## Abstract

**Background**: The COVID-19 pandemic has had a significant impact on the global economy and public health, disrupting various aspects of daily life. Apart from its direct effects on physical health, it has also significantly affected the overall quality of life and mental health. This study employed a path analysis to explore the complex association among multiple factors associated with quality of life, anxiety, and depression in the general population of the Republic of Srpska during the pandemic’s second year. **Method**: A cross-sectional study was conducted on a nationally representative sample (*n* = 1382) of the general population (adults aged 20+) during the second year of the COVID-19 pandemic in the Republic of Srpska, Bosnia, and Herzegovina. Assessment tools included the DASS-21 scale for depression, anxiety, and stress, along with the Brief COPE scale, Quality of Life Scale (QOLS), and Oslo Social Support Scale (OSSS-3). Sociodemographic factors and comorbidities were also assessed. Structural equation modeling was used to identify the direct and indirect links of various characteristics to quality of life, anxiety, and depression. **Results**: This study revealed a considerable prevalence of anxiety and depression symptoms (27.5% and 20.9%, respectively), with quality of life playing a significant mediating role. The constructed path model accounted for 33.1% of moderate to severe depression and 79.5% of anxiety. Negative coping was directly linked to anxiety and indirectly to depression via anxiety, while the absence of positive coping had both direct and indirect paths (through quality of life) on depression. Among variables that directly affected depression, anxiety had the highest effect. However, the bidirectional paths between anxiety and depression were also suggested by the model. **Conclusions**: Pandemic response strategies should be modified to effectively reduce the adverse effects on public mental health. Further research is necessary to assess the long-term effects of the pandemic on mental health and to analyze the contributing factors of anxiety and depression in the post-COVID period.

## 1. Introduction

The emergence of the COVID-19 pandemic has led to a global rise in various factors contributing to poor mental health outcomes. According to the Global Burden of Disease Study, the pandemic significantly increased depression and anxiety rates by 27.6% and 25.6% in 2020, respectively [1]. A recent systematic review identified sociodemographic characteristics (such as gender, age, educational level, and employment status) and comorbidities as significant risk factors associated with depression during this period [2]. These factors included gender, with females being particularly vulnerable, and age, with younger individuals showing a higher risk. Living in urban areas was associated with greater mental health impacts, as was residing in larger households with more than five members. Employment status also played a critical role, with unemployment correlating with an increased risk of mental health problems. Additionally, marital status and educational attainment influenced mental well-being. The presence of children and elderly members in the household further contributed to the psychological burden [2]. Moreover, the stringent policies implemented by countries to curb the pandemic’s spread—including social distancing measures, stricter regulations, and prolonged lockdowns—have taken a toll on the population’s mental health, exacerbating psychological outcomes [3].

In times of crisis, maintaining overall well-being and quality of life can be crucial for protecting mental health. A meta-analysis revealed that the COVID-19 pandemic had a negative effect on the general population’s overall quality of life, where different factors had a role either directly or indirectly [4]. Besides sociodemographic characteristics and living with chronic diseases, confinement and financial constraints affected the quality of life. Moreover, studies have shown a strong link between mental disorders and a decline in overall quality of life [5,6]. Other research has demonstrated that quality-of-life indicators have prognostic implications for mental health outcomes [7,8].

Social support is widely recognized as a critical response mechanism during emergency situations [9]. A meta-analysis of pre-pandemic observational studies indicated that higher perceived levels of social support were associated with a reduced likelihood of endorsing psychiatric symptoms, including depression [10]. It was revealed that received support was strongly correlated with depression [11]. During the COVID-19 pandemic, providing psychosocial support was crucial for mitigating depressive risks among informal caregivers [12]. However, perceptions of the pandemic varied significantly and were influenced by individual psychological and social resilience, which involved diverse coping strategies [13]. Assessing coping strategies, defined as cognitive and behavioral efforts to manage stress in challenging circumstances [14], is essential for understanding the origins of psychological disorders during the pandemic. Research has highlighted a significant association between higher depression levels and avoidance coping styles, as well as lower depression levels and problem-focused coping styles [15,16].

Anxiety and depression are among the most prevalent mental disorders, often presenting with overlapping symptoms and high rates of comorbidity [17]. A global survey indicated that 45.7% of individuals diagnosed with major depressive disorder had also had a history of one or more anxiety disorders in their lifetime, implying the coexistence of both disorders within the same time frame [18]. Apart from their correlation, researchers have explored the causal relationship between anxiety and depression. Studies have identified pathways where anxiety can lead to depression, as well as vice versa [19], while bi-directional relationships have also been proposed [20]. While existing research has established links between anxiety, depression, sociodemographic factors, quality of life, and social support, none have specifically examined the bidirectional pathways linking anxiety and depression during crises like the COVID-19 pandemic. In addition, no studies have yet assessed the prevalence of anxiety and depression and predictors of mental health in the specific context of the Republic of Srpska during the COVID-19 pandemic. Therefore, this study aimed to address these gaps by investigating the complex relationship between quality of life, anxiety, and depression using path analysis, with special emphasis on the existence of bidirectional paths within the general population of the Republic of Srpska during the second year of the COVID-19 pandemic.

## 2. Materials and Methods

### 2.1. Study Design

This cross-sectional study was conducted among the general population (adults aged 20 years and older) during the second year of the COVID-19 pandemic in the Eastern part of the Republic of Srpska, Bosnia, and Herzegovina. Data collection took place during December 2021 and January 2022 within primary healthcare facilities in the situation of lifted containment measures, high incidence of COVID-19 cases, the introduction of vaccination efforts, and significant economic consequences of the pandemic.

### 2.2. Sample

A random stratified sample was used to ensure a statistically reliable assessment of the general population of the Republic of Srpska. In total, 31 municipalities in the eastern part of the Republic of Srpska were included (strata) (Supplemental Table S1). The stratum sample size was determined specifically for each municipality according to its population size. Households/families from each stratum were selected using the linear sampling method with a random start and equal sampling intervals (equal selection steps). The number of households needed for the study was defined according to the number of inhabitants per municipality, taking into account urban and rural households (using a 30:70 ratio of urban vs. rural households). The average household size used for this calculation was 2.91. The sampling frame consisted of a list of families registered with their family doctor in a primary healthcare center in each municipality. All adult residents of selected households were invited to participate. The response rate of participants was 97%. The participation was voluntary and anonymous.

### 2.3. Ethical Considerations

The ethical standards of this study align with international guidelines (Helsinki Declaration). The Law on Personal Data Protection, the Law on Statistics of the Republic of Srpska, the Law on Statistics of Bosnia and Herzegovina, and the European Parliament directive on the protection of personal data guided the privacy of participants and the confidentiality of information. This study received approval from the ethical committee of the Faculty of Medicine in Foča.

### 2.4. Study Instruments

This study utilized sociodemographic questionnaires, questionnaires related to COVID-19 infection, vaccination, and the presence of comorbidities, along with relevant standardized instruments for assessing mental health, social support, quality of life, and coping strategies. Data about the presence and type of comorbidities were self-reported.

In the selection of study instruments, several critical criteria were prioritized to streamline participant engagement and data collection. Firstly, the instruments were chosen for their conciseness, ensuring minimal time commitment for participants. This was vital to enhance response rates and reduce respondent burden. Secondly, no special skills were required to administer questionnaires, i.e., there was no need for additional training. Additionally, the instruments were designed around a single, clear, systematic scale. This uniformity facilitates straightforward data interpretation and consistency across responses. The lack of necessity for permission to use these tools was another significant factor, simplifying the administrative process and expediting the research timeline. Lastly, the scoring system of these instruments could be automated, further enhancing efficiency by enabling quick and accurate data processing. These attributes collectively contributed to an effective and pragmatic approach to instrument selection in this study.

### 2.5. The Depression, Anxiety, and Stress Scale—Short Form (DASS-21)

The DASS-21 scale was used for the assessment of depression, anxiety, and stress in the general population in this study. The DASS-21 scale presents a shorter version of the DASS-42 scale and serves as a standardized self-report tool for assessing unpleasant emotional states or symptoms of depression, anxiety, and stress. It consists of three subscales, each with 7 questions, designed to evaluate states of depression, anxiety, and stress present in the past week. The “Depression” subscale comprises items that assess fundamental depression symptoms, such as low positive affect, dysphoria, hopelessness, devaluation of life, self-deprecation, lack of interest and involvement, anhedonia, and inertia. The “Anxiety” subscale assesses physiological arousal symptoms (dry mouth, difficulty breathing), skeletal muscle effects (trembling), situational anxiety, and the subjective sense of anxious affect. The “Stress” subscale evaluates symptoms of general, nonspecific arousal, such as difficulty relaxing, nervous arousal, ease of becoming upset and agitated, irritability, oversensitivity, and impatience [21].

Participants use a 4-point Likert scale to assess how they felt in the last week, indicating the severity/frequency of depression, anxiety, and stress symptoms they experienced, ranging from 0 (“not at all”) to 3 (“mostly or almost always”). Depression, anxiety, and stress scores are obtained by summing the scores of relevant items in the range of 0–21 and multiplying by 2 for each subscale. The severity of symptoms is categorized using cut-off scores to define normal, mild, moderate, significant, and very significant scores for each subscale. The normal score (absence of symptoms) for the depression subscale ranges from 0 to 9, for anxiety from 0 to 7, and for stress from 0 to 14. Mild symptomatology is defined by scores of 10–13 for the depression subscale, 8–9 for the anxiety subscale, and 15–18 for the stress subscale. Moderate symptomatology is characterized by scores of 14–20 for the depression subscale, 10–14 for the anxiety subscale, and 19–25 for the stress subscale. Severe symptomatology is indicated by scores of 21–27 for the depression subscale, 15–19 for the anxiety subscale, and 26–33 for the stress subscale. Extremely severe symptomatology involves scores > 28 for the depression subscale, >20 for the anxiety subscale, and >34 for the stress subscale [21]. The DASS21 depression subscale demonstrated good internal consistency (α = 0.948) and has been shown to display good discriminant and convergent validity [22]. Additionally, multiple studies have demonstrated construct validity for the subscale in different populations [23,24].

### 2.6. Coping Scale—Short Version

The Coping Scale—Short Version assesses coping strategies individuals use in stressful situations [25]. The questionnaire comprises 28 questions covering the evaluation of 14 strategies: distraction, active coping, denial, substance use, emotional support, instrumental support, behavioral disengagement, venting, positive reinterpretation, planning, humor, acceptance, religion, and self-blame. Participants respond using a four-point Likert scale, ranging from 1 (“do not use at all”) to 4 (“use often”). By summing the points for items within each subscale, total scores for the subscales are obtained. A higher average score on each of the 14 subscales indicates a more frequent application of the respective coping strategy. In 2001, Meyer classified strategies measured by this scale into maladaptive, which include distraction, denial, substance use, behavioral disengagement, venting, and self-blame, and adaptive strategies, which encompass active coping, emotional support, instrumental support, positive reinterpretation, planning, humor, acceptance, and religion [26]. The Coping Scale—Short Version demonstrated good internal consistency (α = 0.935) and has been shown to display good discriminant and convergent validity [27].

### 2.7. Quality of Life Scale

The Quality of Life Scale comprises 16 items assessing the quality of life in five domains: material and physical well-being, relationships with others, social and civic activities, personal development and self-fulfillment, and recreation [28]. Participants respond to the questions using a seven-point Likert scale, ranging from 1 (“completely dissatisfied”) to 7 (“delighted”). The score is obtained by summing all items and can range from 16 to 112, where a higher score indicates a higher quality of life. There is good evidence for the validity and reliability of the Quality of Life Scale, which demonstrates strong construct validity and good content validity [28,29]. The Quality of Life Scale demonstrated good internal consistency in our study (α = 0.939).

### 2.8. Social Support Scale

The Oslo-3 Social Support Scale consists of three questions where a certain number of points are assigned for each answer. The questions are as follows: “How many people are so close to you that you can count on them when you have serious personal problems?” (the number of points ranges from 1 (“None”) to 4 (“6 or more”)). “How much interest and concern do people show in what you do?” (the number of points ranges from 1 (“Not at all interested”) to 5 (“Very interested”)). “How easy is it to get practical help from neighbors if you need it?” (the number of points ranges from 1 (“Very difficult”) to 5 (“Very easy”)). After summing the points, a social support score is formed: strong social support (12–14 points), moderate (9–11 points), and poor (3–8 points) [30]. There is good evidence for the validity and reliability of the Oslo-3 Social Support Scale [30].

### 2.9. Statistical Analysis

Given the complex nature of anxiety and depression, a comprehensive path model was employed to better understand the relationship between different characteristics linked to quality of life, anxiety, and depression within the general population in the Republic of Srpska. Path analysis, a robust statistical method, was chosen for its ability to estimate a system of equations that delineates all possible linkages among a set of variables. In addition, path analysis enables the disintegration of correlations among assessed variables into significant (both direct and indirect) and nonsignificant components. Through this, path analysis effectively disentangles the complex interplay between variables, identifying the most significant pathways within the hypothesized model.

Numerical data are presented as the mean with standard deviation. Categorical variables are summarized with absolute numbers and percentages. The absence of multicollinearity between variables used in the model was confirmed by Pearson’s correlation coefficient, tolerance, and variance inflation factor (VIF) before the assessment of direct and indirect paths. All estimates were within acceptable ranges: r < 0.8, tolerance ≥ 0.1, and VIF ≤ 10. The skewness and kurtosis coefficients for continuous variables were below 1. The adequacy of model-fit to the data was determined by multiple measures, including the χ^2^ test and the following fit indices: the root mean square error of approximation (RMSEA), the comparative fit index (CFI), the Tucker–Lewis index (TLI), and the Incremental fit index (IFI). These fit indices were used to indicate the degree to which a pattern of fixed and free parameters specified in the model was consistent with the pattern of variances and covariances from a set of observed data. The model is presented graphically where the arrows demonstrate the direction of the hypothesized association. The strength of the path between variables is presented by standardized regression coefficients. To enable comparison between variables, the standardized effects were estimated to illustrate path coefficients on a standardized scale ranging from −1 to 1. The direct coefficients present the effect of an independent variable on a dependent variable after controlling for other predictors in the model, while the indirect coefficients demonstrate the effect of an independent variable on a dependent variable, which is mediated by variables along the path. The total effect is calculated as the sum of the direct and indirect effects. In all analyses, the significance level was set at 0.05. Statistical analysis was conducted using Amos 21 (IBM SPSS Inc., Chicago, IL, USA, 2012) and IBM SPSS Statistics 25 software.

## 3. Results

This study included a total of 1382 participants from the Eastern part of the Republic of Srpska. The majority of participants were female (60.0%) and aged between 40 and 59 years (41.8%). Half of the participants were employed (50.5%), and 59.0% had a secondary education. More than half of the tested participants were PCR positive (57.8%), and 64.5% had been vaccinated. Regarding pre-existing comorbidities, the highest percentage of participants had hypertension (32.1%), obesity (25.4%), and elevated cholesterol levels (18.9%). Sociodemographic, COVID-specific characteristics, and comorbidities are presented in Table 1.

Every fifth participant exhibited some form of depression (20.9%) and stress (21.0%), while almost every third participant experienced some form of anxiety (27.5%). The average quality of life score was 81.88 ± 19.29, and most of the participants had poor social support (90.7%). Regarding coping mechanisms used, the general population most frequently utilized acceptance (5.19), emotional support (4.95), instrumental support (4.94), active coping (4.91), and humor (4.88), while substance use was the least utilized coping mechanism (2.58) (Table 2).

**Figure 1:**
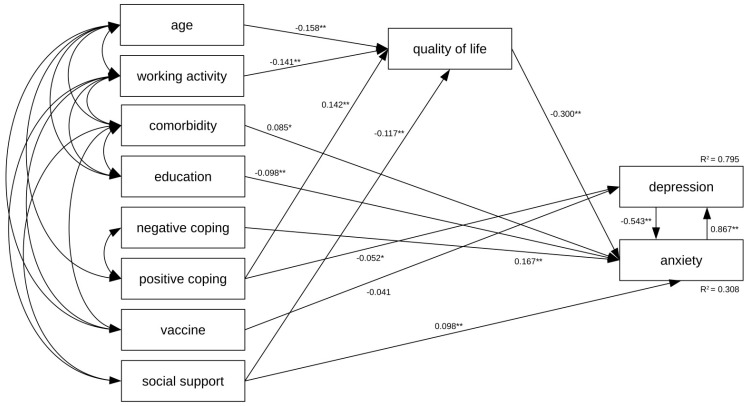
Path model presenting the complex relationship between assessed variables linked to anxiety and depression. * *p* < 0.05; ** *p* < 0.01.

The hypothesized relationships among the assessed variables were tested by path analysis using a maximum likelihood estimate. Path analysis was used as it allowed the assessment of the direct and indirect effects of different characteristics on outcomes through simultaneous modeling of related regression relationships. The model with depression as the main outcome accounted for 37.4% of moderate to severe depression (Supplemental Figure S1). The best fit of the path model was achieved with χ^2^ = 70.303, df = 30, CMIN/DF = 2.343, *p* < 0.001, TLI = 0.969, IFI = 0.983, CFI = 0.983, and RMSEA =0.031. According to this model, age, working activity, positive coping, and social support were directly linked to quality of life. Decreased quality of life and negative coping, as well as lower level of education, presence of comorbidities, and low social support, were directly linked to anxiety. Increased anxiety, the absence of positive coping, and vaccination were directly linked to depression. Among variables that directly affected depression, anxiety had the highest effect, and vaccination had the lowest effect (Supplemental Table S2). It is interesting to note that negative coping was correlated to depression via anxiety, while the absence of positive coping had both direct and indirect paths (through quality of life) on depression (Supplemental Table S2). In addition, the path between depression and anxiety was added and evaluated. This modified path model presenting bidirectional effects between anxiety and depression is presented in Figure 1. The modified model accounted for 33.1% of moderate to severe depression and 79.5% of anxiety. The fit indexes for the modified bidirectional model were TLI = 0.976, IFI = 0.988, CFI = 0.988, and RMSEA = 0.027; all met the suggested benchmarks and are improved over the original model. The lower value of AIC for the modified model indicates a better fit to the hypothesized model in contrast to the original model (154.303 vs. 164.303). According to this model, depression also had a direct link to anxiety, and among variables that directly affected anxiety, depression had the highest effect, but in the negative direction (B = −0.543), while the presence of comorbidities (B = −0.085) had the lowest effect. The absence of positive coping and vaccination had significant indirect paths to anxiety via depression (Supplemental Table S3).

## 4. Discussion

The COVID-19 pandemic has had a significant impact on the global economy and public health, disrupting various aspects of daily life. Apart from its direct effects on physical health, it has also significantly affected the overall quality of life and well-being.

This study employed a path analysis to explore the complex association among multiple factors associated with quality of life, anxiety, and depression in the general population of the Republic of Srpska during the pandemic’s second year. This study revealed a considerable prevalence of anxiety and depression symptoms, with quality of life playing a significant mediating role. Approximately one in five participants (20.9%) reported experiencing some degree of depression, ranging from mild to extremely severe. Almost a third of the participants experienced some form of anxiety (27.5%).

Numerous studies on mental health have reported varying rates of mental disorders during the COVID-19 pandemic. Some of these discrepancies can be attributed to methodological issues, such as different measurement approaches and cut-off scores [31]. Other differences likely stem from cultural factors that influence the identification and reporting of mental health problems. Furthermore, variations were also influenced by the timing of mental health data collection, as the stringency of governmental measures to curb the spread of COVID-19 fluctuated over time and across countries [32]. In a recent umbrella meta-review, the prevalence of anxiety symptoms ranged from 24.4% in general populations to 41.1% in vulnerable populations. The prevalence of depressive symptoms ranged from 22.9% in general populations to 32.5% in vulnerable populations. The same study revealed standardized mean differences in pre-COVID-19 to during COVID-19 prevalence of depression and anxiety of 0.20 (95% CI = 0.07–0.33) and 0.29 (95% CI = 0.12–0.45), respectively [33]. A study conducted in Serbia [34] on a sample of the general population during the initial phase of the pandemic reported nearly double the rates of depression (42%) and anxiety (44.5%). These high levels of depression and anxiety were likely driven by the implementation of quarantine and lockdown measures.

In our study, the majority of participants (60.0%) were women, with the largest age group being between 40 and 59 years old (41.8%). About half of the participants (50.5%) were employed, and slightly over half (59.0%) had completed secondary education. Similar sociodemographic patterns were observed in studies conducted in Switzerland [35] and the United States [36]. In Switzerland, factors associated with psychological distress included younger age, female gender, single parenthood, unemployment, recent changes in employment status, and higher concerns about COVID-19 severity and contagiousness. In the United States, a study analyzed predictors of mental health in adults eight months after the pandemic began. This study highlighted differences in mental health based on gender, race, age, employment status, and levels of concern regarding COVID-19. The comparisons made in this study underscore variations in demographic characteristics and mental health outcomes across different populations during the COVID-19 pandemic.

In our study population, 57.8% of participants tested positive on PCR tests. A study conducted by U.S. researchers reported a slightly higher percentage (72.2%) of positive COVID tests among participants, assessing the physical, mental health, and social well-being of COVID-positive and COVID-negative patients three months post-diagnosis [37]. This study also noted prevalent comorbidities, with obesity at 30.0% and hypertension at 18.1% among respondents. In our study, obesity was similarly prevalent at 25.4%, while hypertension was slightly higher at 32.1%. Bonati and colleagues’ systematic literature review at the pandemic’s onset highlighted sociodemographic factors (such as age, income level, and employment status) and pre-existing chronic conditions as significant risk factors for depression and anxiety in Europe’s adult population [38]. In our study, these factors exerted an indirect effect, with age and employment status affecting quality of life and education and comorbidities impacting anxiety.

Vaccination emerged to have a significant direct link to depression in our study. However, the literature on the relationship between COVID-19 vaccination and mental health presents varied results. While studies generally indicate that vaccination improves mental health by reducing the likelihood of depression and anxiety symptoms [39,40], a recent meta-analysis concluded that COVID-19 vaccination itself does not show a clear association with depression and anxiety [41]. In contrast, a longitudinal study encompassing 28,293 adult residents of Sweden highlighted the importance of the number of vaccine doses received in the context of mental health. Specifically, this study found a significant reduction in the prevalence of depressive and anxiety symptoms, particularly after the administration of the second vaccine dose [42].

In our study, most respondents in the second year of the pandemic reported high scores for quality of life, which aligns with previous research indicating a notable improvement in quality of life as the pandemic has evolved. This positive trend is largely linked to the easing of restrictions and overall situation stabilization [43,44]. A population-based study in Germany demonstrated that older age and income loss predicted reduced quality of life during the pandemic’s second year [43]. Quality of life emerged as a crucial factor in our study, playing both a direct and mediating role in depression onset. This finding is supported by an Italian study that utilized path modeling to explain the pivotal role of quality of life in the mental health of the general population during the pandemic [44]. Their results indicated that as participants’ fear of COVID-19 infection decreased between waves of the pandemic, negative mental states such as stress, anxiety, and depression also decreased, thereby enhancing perceived quality of life. Quality of life was found to mitigate the impact of COVID-19 fears on participants’ mental well-being over both short and long terms, underscoring its critical role in regulating mental health.

While a meta-analysis by Spanish authors suggested a modest association between social support and symptoms of mental disorders during the COVID-19 pandemic [45], our study revealed that social support indirectly influenced depression through its mediation of both quality of life and anxiety. However, there was a stark contrast as 90.7% reported poor social support in our study. Despite employing a similar methodology to assess social support levels, our findings differ from those of a French study where only 17.0% of respondents reported poor social support [46]. The significant prevalence of poor social support in our population during the pandemic’s second year may be linked to the direct impact of the pandemic itself, alongside socioeconomic and cultural influences. Moisoglou et al., in a cross-sectional study of individuals with post-COVID syndrome, found a positive association between social support, quality of life, and mental health, emphasizing social support’s protective role in reducing anxiety and depression while improving overall quality of life [47]. Additionally, research conducted in France at the pandemic’s initial peak indicated that individuals with lower levels of education faced a higher risk of developing anxiety and depression, consistent with our own findings [48].

A recent study highlighted coping strategies as significant predictors of mental health outcomes during the COVID-19 pandemic [49]. In a Portuguese study, the absence of positive coping mechanisms and the presence of negative mechanisms were associated with higher levels of depression and anxiety, aligning with the findings of our study [50]. Commonly employed coping mechanisms included positive strategies such as acceptance, active coping, positive reinterpretation, and planning [51]. A study from Malaysia demonstrated that the most frequently employed coping strategies among the adult population were active coping and humor [52]. These findings align with the results of our study, suggesting a consistent pattern in coping mechanisms across different populations. The association between negative coping mechanisms and the prevalence of anxiety and depression has been corroborated by studies conducted by Spanish and Italian researchers [53,54]. An American study revealed that positive coping mechanisms, specifically problem-focused and emotion-focused coping, were significantly associated with higher levels of quality of life [55]. This finding is consistent with the results of our study. However, the same study also found that negative coping mechanisms, such as avoidant coping, were associated with lower quality of life. However, this particular association was not confirmed in our study.

Our study revealed that among the factors examined, anxiety exerted the strongest direct link to depression. This finding is consistent with research by Rodriguez-Hidalgo et al., which highlighted anxiety’s significant mediating role in depression among students during the pandemic [51]. In addition to exploring the associations between anxiety and depression, researchers have investigated the causal relationship between these two conditions. Jacques and Mash utilized structural equation modeling to uncover that anxiety can lead to depression, and conversely, depression can contribute to anxiety. Subsequent research by Dia et al. proposed a reciprocal relationship, suggesting that anxiety influences depression and vice versa [19,20]. Our modified model supports the bidirectional relationship between anxiety and depression in the general population during the COVID-19 pandemic. Several evidence-based recommendations aimed at enhancing community well-being and resilience in future pandemics could be proposed based on the results of our study and the literature review [56]. These recommendations should focus on proactive measures that extend beyond clinical treatment, emphasizing mental health promotion and adoption of protective behaviors. Prioritizing these actions can empower communities to maintain well-being and resilience in the face of challenges posed by public health emergencies such as the COVID-19 pandemic.

Effective public health education initiatives must disseminate tailored information on mental health risks and protective behaviors while dispelling misinformation about COVID-19. Regular exercise, open communication within families, and adherence to daily routines—such as maintaining a balanced diet, ensuring adequate sleep, participating in social activities, pursuing hobbies, and fulfilling work and study commitments—are essential for mental health.

When formulating policies on social distancing and quarantine, it is crucial for countries to consider not just epidemiological conditions but also the mental health of their populations. Overly uncompromising and strict policies have been associated with increased depression rates, highlighting the need for balanced approaches that address comprehensive health impacts.

Leveraging technology, such as mobile apps and wearables, can enhance mental health interventions and enable real-time monitoring of community well-being. Mobile apps can provide resources for self-care, well-being assessments, and data tracking, informing targeted interventions and resource allocation in response to evolving mental health needs during emergencies.

Furthermore, the COVID-19 pandemic has underscored disparities and financial challenges in healthcare systems, particularly in middle- and low-income countries. Recommending investment in universal health coverage is critical to ensuring equitable access to healthcare regardless of socioeconomic status.

Integrating routine screening programs for anxiety and depression into primary healthcare practices in the Republic of Srpska should be imperative. This approach reduces barriers to the prevention, identification, and management of depression.

Overall, recommended strategies should be clearly relevant and provide practical guidance to empower individuals, families, and community leaders in safeguarding public well-being.

This study has several limitations. Cross-sectional studies collect data at one point in time; thus, the use of a cross-sectional design restricts the ability to establish causal relationships or determine temporal associations between variables. This design only allows for the observation of associations or correlations, not causality. Since cross-sectional studies measure all variables simultaneously, it is impossible to discern the temporal sequence of events. This lack of temporal data hinders the ability to determine which variable precedes the other. In addition, cross-sectional studies may suffer from survivorship bias, as they only include individuals who are present at the time of the study. Those who may have been affected differently or dropped out due to severe conditions or mortality are not represented, potentially skewing results. The assessment of social support relied on the availability of individuals who could provide assistance during the epidemic, potentially underestimating its actual influence. Future research should incorporate more comprehensive measures to better understand the impact of social support on depressive symptoms. Vaccination status in our study was self-reported, which may introduce recall bias. Further research is warranted to comprehensively assess the impact of COVID-19 vaccination on depression, especially given the diversity of available vaccines in the market.

## 5. Conclusions

This study revealed a considerable prevalence of anxiety and depression symptoms in the general population of the Republic of Srpska during the second year of the COVID-19 pandemic. Quality of life served a significant mediating role and hypothesized bidirectional pathways between anxiety and depression were confirmed by the model. A multidisciplinary approach is critical in devising strategies to uphold and improve mental health and prevent mental disorders in the adult population of the Republic of Srpska.

The findings of this study enable the identification of vulnerable groups in need of psychological counseling and social connections to reduce the burden of psychiatric disorders such as depression and anxiety. These findings can also inform health policies to implement mental health interventions such as screenings in primary healthcare institutions, highlighting the need for increased investment and access to mental health services. Further research is necessary to assess the long-term effects of the pandemic on mental health and to analyze the contributing factors of anxiety and depression in the post-COVID period.

## Figures and Tables

**Table 1:** Sociodemographic and COVID-specific characteristics and comorbidities.

Variables	n	%
Gender		
Male	553	40.0
Female	829	60.0
Age groups		
20–39	339	24.8
40–59	571	41.8
≥60	455	33.3
Average age ($\overset{–}{x}$ ± SD)	50.6 ± 14.7	
Level of education		
Primary education	163	12.1
Secondary education	796	59.0
Tertiary education	391	29.0
Employment status		
Employed	698	50.5
Unemployed	231	16.7
Retired	420	30.4
Other	33	2.4
COVID specific characteristics		
Tested by PCR test	550	40.0
PCR test positive	318	57.8
Vaccinated against COVID-19	868	64.5
Comorbidities		
Cardiovascular disease	162	11.8
Hypertension	440	32.1
Cerebrovascular disease	16	1.2
Malignant disease	40	2.9
Hormonal imbalance	52	3.8
Diabetes mellitus	131	9.5
Chronic obstructive pulmonary disease	44	3.2
Chronic kidney disease	22	1.6
Chronic liver disease	11	0.8
Autoimmune disease	48	3.5
Other diseases and conditions	50	3.6
Obesity	349	25.4
Hypercholesterolemia	259	18.9

**Table 2:** Mental health, quality of life, social support, and coping strategies.

Variables	n	%
Mental health		
Depression	288	20.9
Anxiety	380	27.5
Stress	289	21.0
Quality of life, mean ± sd	81.88 ± 19.29	
Social support		
Poor	1249	90.7
Moderate	124	9.0
Strong	4	0.3
Coping strategies	mean ± sd	
Distraction	4.57 ± 1.87	
Active coping	4.91 ± 2.02	
Denial	3.41 ± 1.63	
Substance use	2.58 ± 1.24	
Emotional support	4.95 ± 1.99	
Instrumental support	4.94 ± 1.84	
Behavioral disengagement	3.05 ± 1.47	
Venting	3.74 ± 1.58	
Positive reframing	4.86 ± 1.96	
Planning	4.22 ± 1.67	
Humor	4.88 ± 2.09	
Acceptance	5.19 ± 1.99	
Religion	4.02 ± 1.97	
Self-blame	3.67 ± 1.59	

## Data Availability

Data are contained within this article or Supplementary Materials.

## Supplementary Materials

The following supporting information can be downloaded at https://www.mdpi.com/article/10.3390/jcm13133874/s1, Table S1. Study population distribution by city/municipality. Table S2. Direct, indirect, and total effects on depression. Table S3. Direct, indirect, and total effects on anxiety and depression. Figure S1. Path model presenting the complex relationship between assessed variables linked to depression.
