# Supplementary material for: A Complex Relationship between Quality of Life, Anxiety, and Depression among General Population during Second Year of COVID-19 Pandemic: A Population-Based Study
Source: J Clin Med. 2024 Jun 30;13(13):3874. doi: 10.3390/jcm13133874 (PMC11242713; doi:10.3390/jcm13133874)
Supplement: Supplementary file 1 [file jcm-13-03874-s001.zip › jcm-3039946-supplementary.pdf]

**Supplemental Table S1.** Study population distribution by city/municipality

| City/municipality | Total number of households | Number of households included in the study | Number of participants included in the study (N) |
|---|---|---|---|
| Banja Luka | 65010 | 268 | 740 |
| **Berkovići** | **636** | **3** | **8** |
| **Bijeljina** | **34309** | **141** | **427** |
| **Bileća** | **3307** | **14** | **44** |
| **Bratunac** | **5945** | **25** | **77** |
| Brod | 5599 | 23 | 65 |
| **Višegrad** | **3966** | **16** | **41** |
| **Vlasenica** | **3712** | **15** | **44** |
| Vukosavlje | 1537 | 6 | 18 |
| **Gacko** | **2766** | **11** | **36** |
| Gradiška | 16939 | 70 | 203 |
| Derventa | 9345 | 39 | 107 |
| **Doboj** | 25447 | 105 | 281 |
| Donji Žabar | 1191 | 5 | 15 |
| **Zvornik** | **17690** | **73** | **224** |
| Istočni Drvar | 38 | 0 | 0 |
| **Istočni Mostar** | **83** | **0** | **1** |
| **Istočna Ilidža** | **4981** | **21** | **59** |
| **Istočni Stari Grad** | **438** | **2** | **5** |
| **Istočno Novo Sarajevo** | **3819** | **16** | **43** |
| **Pale** | **7158** | **30** | **84** |
| **Sokolac** | **4276** | **18** | **48** |
| **Trnovo** | **800** | **3** | **8** |
| Jezero | 365 | 2 | 4 |
| **Kalinovik** | **783** | **3** | **8** |
| Kneževo | 3168 | 13 | 38 |
| Kozarska Dubica | 7492 | 31 | 85 |
| Kostajnica | 1896 | 8 | 23 |
| Kotor Varoš | 5863 | 24 | 76 |
| Krupa na Uni | 543 | 2 | 6 |
| Kupres | 128 | 1 | 1 |
| Laktaši | 11293 | 47 | 141 |

| | | | |
|---|---|---|---|
| **Lopare** | **5081** | **21** | **60** |
| **Ljubinje** | **1077** | **4** | **14** |
| **Milići** | **3579** | **15** | **43** |
| Modriča | 8553 | 35 | 100 |
| Mrkonjić Grad | 6104 | 25 | 65 |
| **Nevesinje** | **3985** | **16** | **52** |
| Novi Grad | 8792 | 36 | 104 |
| **Novo Goražde** | **1078** | **4** | **12** |
| **Osmaci** | **1675** | **7** | **23** |
| Oštra Luka | 971 | 4 | 11 |
| Pelagićevo | 1787 | 7 | 18 |
| Petrovac | 130 | 1 | 1 |
| Petrovo | 2532 | 10 | 26 |
| **Prijedor** | 27961 | 115 | 332 |
| Prnjavor | 12166 | 50 | 141 |
| Ribnik | 2252 | 9 | 24 |
| **Rogatica** | **3871** | **16** | **42** |
| **Rudo** | **2847** | **12** | **31** |
| Srbac | 6157 | 25 | 70 |
| **Srebrenica** | **3792** | **16** | **48** |
| Teslić | 13255 | 55 | 154 |
| Trebinje | 9394 | 39 | 116 |
| Ugljevik | 4867 | 20 | 62 |
| Foča | 6544 | 27 | 71 |
| Han Pijesak | 1390 | 6 | 14 |
| Čajniče | 1662 | 7 | 19 |
| Čelinac | 5009 | 21 | 62 |
| Šamac | 5609 | 23 | 67 |
| **Šekovići** | **2517** | **10** | **26** |
| Šipovo | 3665 | 15 | 41 |
| **TOTAL** | **408,825** | **1686** | **4812** |

Bolded municipalities: eastern part of the Republic of Srpska

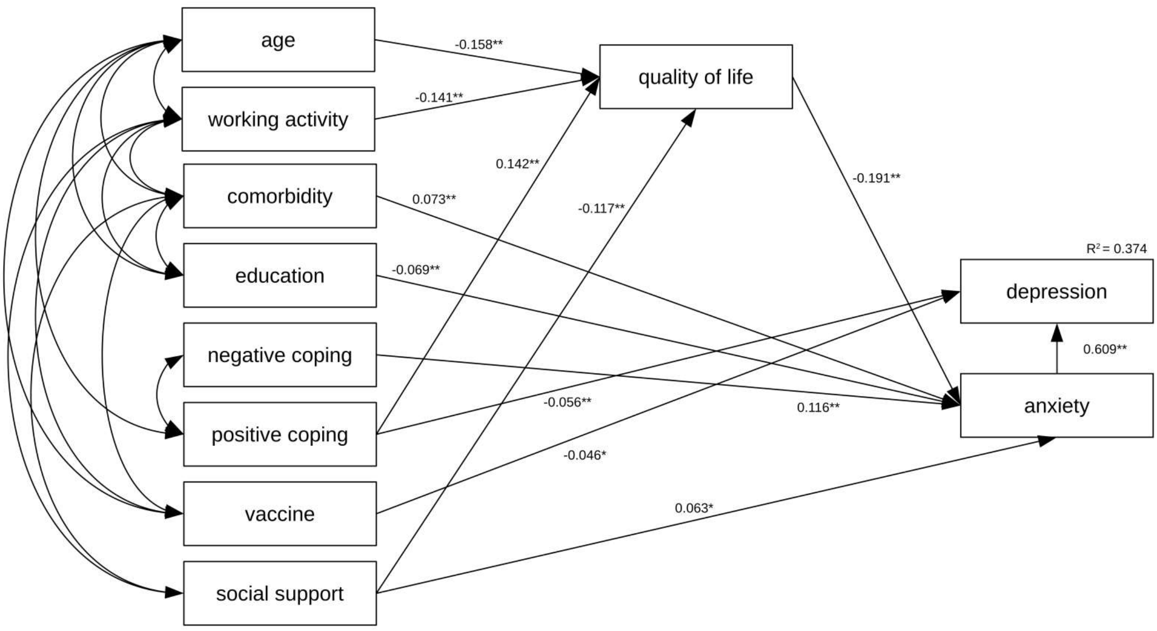


**Supplemental Figure S1.** Path model presenting the complex relationship between assessed variables linked to depression.

**Supplemental Table S2.** Direct, indirect, and total effects on depression.

| Variables | B | SE | *p* |
|---|---|---|---|
| **Direct** | | | |
| Vaccine → Depression | -0.046 | 0.022 | 0.018 |
| Positive Coping → Depression | -0.056 | 0.021 | 0.007 |
| Anxiety → Depression | 0.609 | 0.026 | 0.001 |
| Age → QoL | -0.158 | 0.029 | 0.001 |
| Working activity → QoL | -0.141 | 0.031 | 0.001 |
| Positive Coping → QoL | 0.142 | 0.024 | 0.002 |
| Social Support → QoL | -0.117 | 0.028 | 0.001 |
| Education→ Anxiety | -0.069 | 0.024 | 0.004 |
| Comorbidities → Anxiety | 0.073 | 0.027 | 0.008 |
| Negative Coping → Anxiety | 0.116 | 0.029 | 0.001 |
| Social support → Anxiety | 0.063 | 0.027 | 0.011 |
| QoL → Anxiety | -0.191 | 0.025 | 0.001 |
| **Indirect** | | | |
| Age → Depression | 0.018 | 0.004 | 0.001 |
| Working activity → Depression | 0.016 | 0.004 | 0.001 |
| Comorbidities → Depression | 0.044 | 0.016 | 0.008 |
| Education → Depression | -0.042 | 0.015 | 0.004 |
| Positive Coping → Depression | -0.016 | 0.004 | 0.001 |
| Negative Coping → Depression | 0.071 | 0.018 | 0.001 |
| Social Support → Depression | 0.052 | 0.017 | 0.001 |
| QoL → Depression | -0.116 | 0.017 | 0.001 |
| Age → Anxiety | 0.030 | 0.007 | <0.001 |
| Working activity → Anxiety | 0.029 | 0.007 | 0.001 |
| Positive Coping → Anxiety | -0.027 | 0.006 | 0.001 |
| Social Support → Anxiety | 0.022 | 0.006 | 0.001 |
| **Total** | | | |
| Age → Depression | 0.018 | 0.004 | 0.001 |
| Vaccine → Depression | -0.046 | 0.022 | 0.018 |
| Working activity → Depression | 0.016 | 0.004 | 0.001 |
| Comorbidities → Depression | 0.044 | 0.016 | 0.008 |
| Education → Depression | -0.042 | 0.015 | 0.004 |
| Positive Coping → Depression | -0.072 | 0.021 | 0.001 |
| Negative Coping → Depression | 0.071 | 0.018 | 0.001 |
| Social Support → Depression | 0.052 | 0.017 | 0.001 |
| QoL → Depression | -0.116 | 0.017 | 0.001 |
| Anxiety → Depression | 0.609 | 0.026 | 0.001 |

**Supplemental Table S3.** Direct, indirect, and total effects on anxiety and depression.

| Variables | B | SE | *p* |
|---|---|---|---|
| **Direct** | | | |
| Vaccine → Depression | -0.041 | 0.023 | 0.073 |
| Positive Coping → Depression | -0.052 | 0.023 | 0.028 |
| Anxiety → Depression | 0.867 | 0.089 | 0.001 |
| Age → QoL | -0.158 | 0.029 | 0.001 |
| Working activity → QoL | -0.141 | 0.031 | 0.001 |
| Positive Coping → QoL | 0.142 | 0.024 | 0.001 |
| Social Support → QoL | -0.117 | 0.028 | 0.001 |
| Education→ Anxiety | -0.098 | 0.037 | 0.005 |
| Comorbidities → Anxiety | 0.085 | 0.039 | 0.037 |
| Negative Coping → Anxiety | 0.167 | 0.053 | 0.001 |
| Social support → Anxiety | 0.098 | 0.043 | 0.007 |
| QoL → Anxiety | -0.300 | 0.074 | 0.001 |
| Depression → Anxiety | -0.543 | 0.281 | 0.004 |
| **Indirect** | | | |
| Age → Depression | 0.028 | 0.007 | 0.001 |
| Working activity → Depression | 0.025 | 0.007 | 0.001 |
| Comorbidities → Depression | 0.050 | 0.022 | 0.037 |
| Education → Depression | -0.058 | 0.020 | 0.005 |
| Positive Coping → Depression | -0.008 | 0.010 | 0.309 |
| Negative Coping → Depression | 0.099 | 0.026 | 0.001 |
| Social Support → Depression | 0.078 | 0.024 | 0.001 |
| QoL → Depression | -0.177 | 0.028 | 0.001 |
| Depression → Depression | -0.320 | 0.119 | 0.004 |
| Anxiety → Depression | -.0278 | 0.136 | 0.004 |
| Age → Anxiety | 0.032 | 0.007 | 0.001 |
| Working activity → Anxiety | 0.029 | 0.007 | 0.001 |
| Comorbidities → Anxiety | -0.027 | 0.019 | 0.040 |
| Positive Coping → Anxiety | -0.010 | 0.011 | 0.309 |
| Social Support → Anxiety | -0.007 | 0.025 | 0.721 |
| Vaccine → Anxiety | 0.015 | 0.009 | 0.077 |
| Anxiety→ Anxiety | -0.320 | 0.119 | 0.004 |
| Depression → Anxiety | 0.174 | 0.191 | 0.001 |
| **Total** | | | |
| Age → Depression | 0.028 | 0.007 | 0.001 |
| Working activity → Depression | 0.025 | 0.007 | 0.001 |
| Comorbidities → Depression | 0.050 | 0.022 | 0.037 |
| Education → Depression | -0.058 | 0.020 | 0.005 |
| Positive Coping → Depression | -0.060 | 0.017 | 0.001 |
| Negative Coping → Depression | 0.099 | 0.026 | 0.001 |
| Social Support → Depression | 0.078 | 0.024 | 0.001 |
| QoL → Depression | -0.177 | 0.028 | 0.001 |
| Anxiety → Depression | 0.589 | 0.049 | 0.001 |
| Depression → Depression | -0.320 | 0.119 | 0.004 |
| Age → Anxiety | 0.032 | 0.007 | 0.001 |
| Working activity → Anxiety | 0.029 | 0.007 | 0.001 |
| Comorbidities → Anxiety | 0.058 | 0.027 | 0.037 |
| Education → Anxiety | -0.066 | 0.023 | 0.005 |
| Positive Coping → Anxiety | -0.010 | 0.011 | 0.309 |
| Negative Coping → Anxiety | 0.114 | 0.028 | 0.001 |
| Social Support → Anxiety | 0.090 | 0.027 | 0.001 |
| QoL → Anxiety | -0.204 | 0.024 | 0.001 |
| Depression → Anxiety | -0.369 | 0.100 | 0.004 |
| Anxiety→ Anxiety | -0.320 | 0.119 | 0.004 |
